# 

**Published:** 1817-01

**Authors:** 


### JOURNAL AND REVIEW.

CONTAINING,

I. Select Original Communications.

II. Comprehensive Analytical Review of all important Medical Publications; presenting a fair Epitome of the Medical Literature of the Day.

III. Select Translations from Foreign Medical Journals.

IV. Medical Miscellanies, Foreign and Domestic; Anonymous Communications, &c. &c.

CONDUCTED BY

WILLIAM SHEARMAN, M. D.

Licentiate of the Royal College of Physicians, and Physician to the London Dispensary;

JAMES JOHNSON, M. D.

Surgeon to His Royal Highness the Duke of Clarence's Household; and Author of "The Influence of Tropical Climates on European Constitutions."

AND

SHIRLEY PALMER, M. D.

Of the University of Glasgow.

OMNIA VINCIT LABOR SI LABOR IPSE VOLUPTAS.

VOL. III.

FROM JANUARY TO JUNE, 1817.

63386

LONDON.

PRINTED FOR THE PROPRIETORS,

BY WILLIAM THORNE, RED LION COURT, FLEET STREET,

To whom Communications, addressed to the Editors, are requested to be sent.

Sold by BALDWIN, CRADOCK, and JOY, Pater-Noster Row; HIGHLEY and SON, and T. & G. UNDERWOOD, Fleet Street; J. CALLOW, Crown Court, Soho; COX and SON, Borough; ANDERSON and CHASE, West Smithfield; W. BLACKWOOD, Edinburgh; J. CUMMING, Dublin; and may be had also by applying to the Clerks of the Roads, at the General Post Office.

### CORRESPONDENCE.

We return thanks to Mr. Coley, of Cheltenham, for his interesting document relative to Vaccination in Java. It is highly creditable to the philanthropy of Mr. Ruffles, the late governor, and must be truly gratifying to Dr. Jenner, whose name is now, by a classical and ingenious device, identified, as it were, with the very *language* of an island containing four millions of inhabitants. It shall have early insertion.— Mr. Coley's favours will always be esteemed.

Further Communications have been received from Dr. Kennedy. The Editors beg leave to thank that gentleman for his judicious hints, and for the interest he takes in their Journal.

The Report of a mysterious Case of Fracture of the Basis Cranii, terminating in inflammation and fatal effusion; with the result of a judicial examination of the body, and the opinions thereupon, officially delivered by Dr. Palmer and Mr. Ward, will shortly appear.

An Essay on Syphiloid Diseases, by a Physician (formerly a pupil at the Lock Hospital) is received, and will appear as soon as room can be spared.

Observations on the Utility of Evacuations and very low Regimen in the treatment and development of obscure diseases with anomalous symptoms, are received.

Oriental Method of curing Rheumatism, with a sketch of the instruments used in that process, is received, and will be attended to.

Dr. Thomson's Report of Military Hospitals in Belgium is reviewed, and waiting insertion.

*A young but coastant Reader's* Letter has been received. We think the object in view would be best attained by the assistance of some professional friend, whose knowledge of the particular bent and inclinations of the party might enable him to enter into those details which no general plan, however elaborate, could embrace. It is probable, however, that we may advert to the subject when we have more leisure to do it justice than we have at present.

---

### *Diseases and Casualties in London in the Year* 1816.

| | |
|---|---|
| Abortive and Stillborn | 734 |
| Abscess | 106 |
| Aged | 1913 |
| Ague | 3 |
| Apoplexy & Suddenly | 434 |
| Afthma | 1003 |
| Bedridden | 5 |
| Bile | 1 |
| Bleeding | 30 |
| Burften and Rupture | 35 |
| Cancer | 79 |
| Chicken Pox | 1 |
| Childbed | 234 |
| Colds | 19 |
| Colic, Gripes, &c. | 6 |
| Confumption | 4272 |
| Convulfions | 3264 |
| Cough, and Hooping Cough | 666 |
| Cramp | 2 |
| Croup | 92 |
| Diabetes | 5 |
| Dropfy | 788 |
| Dysentery | 1 |
| Epilepfy | 4 |
| Evil | 8 |
| Fevers of all kinds | 1299 |
| Fiftula | 8 |
| Flux | 15 |
| French Pox | 61 |
| Gout | 56 |

| | |
|---|---|
| Gravel, Stone, and Strangury | 14 |
| Grief | 4 |
| Headmoldfhot, Horsefhoe head, and water in the head | 408 |
| Inflammation | 977 |
| Jaundice | 76 |
| Jaw Locked | 2 |
| Leprosy | 1 |
| Lethargy | 1 |
| Livergrown | 79 |
| Lunatick | 230 |
| Meafles | 1106 |
| Mifcarriage | 7 |
| Mortification | 327 |
| Palpitation of the Heart | 11 |
| Palfy | 195 |
| Pleurify | 22 |
| Purples | 2 |
| Quinfy | 2 |
| Rafh | 1 |
| Rheumatifm | 14 |
| Rifing of the Lights | 1 |
| Scrophula | 2 |
| Scurvy | 2 |
| Shingles | 1 |
| Small Pox | 653 |
| Sore Throat | 13 |
| Sores and Ulcers | 15 |
| Spafm | 43 |

| | |
|---|---|
| St. Anthony's Fire | 7 |
| Stoppage in the Stom. | 26 |
| St. Vitus's Dance | 1 |
| Swelling | 2 |
| Swine Pox | 1 |
| Teeth | 417 |
| Thrufh | 89 |
| Tumor | 3 |
| Water in the Cheft | 48 |
| Worms | 15 |
| Broken Limbs | 3 |
| Burnt | 48 |
| Drowned | 105 |
| Exceffive Drinking | 13 |
| Executed | 10 |
| Found Dead | 31 |
| Fractured | 4 |
| Frighted | 6 |
| Killed by Falls and feveral other Accidents | 56 |
| Killed by Fighting | 1 |
| Killed themfelves | 50 |
| Murdered | 8 |
| Overlaid | 2 |
| Poifoned | 8 |
| Scalded | 5 |
| Suffocated | 3 |
| Total | 354 |

| | | | |
|---|---|---|---|
| Chriftened | Males | 12132 | In all 23581 |
| | Females | 11449 | |
| Buried | Males | 10105 | In all 20316 |
| | Females | 10211 | |

Whereof have died,

| | | | |
|---|---|---|---|
| Under Two Years of Age | 5400 | Sixty and Seventy | 1720 |
| Between Two and Five | 1960 | Seventy and Eighty | 1308 |
| Five and Ten | 845 | Eighty and Ninety | 781 |
| Ten and Twenty | 675 | Ninety and a Hundred | 168 |
| Twenty and Thirty | 1464 | A Hundred | 3 |
| Thirty and Forty | 1912 | A Hundred and One | 0 |
| Forty and Fifty | 2123 | A Hundred and Three | 1 |
| Fifty and Sixty | 1955 | A Hundred and Four | 1 |

Increafed in the Burials this Year 756.

